# Sentinel lymph node biopsy in cN0 neck management of parotid cancer

**DOI:** 10.1038/s41598-019-44790-3

**Published:** 2019-06-06

**Authors:** Wenting Pan, Kai Ba, Xingyu Niu, Fei Liu, Qigen Fang

**Affiliations:** 1 Stomatology Center, The First Affiliated Hospital of Zhengzhou University, Zhengzhou, P.R. China; 2 Department of Head and Neck, Affiliated Cancer Hospital of Zhengzhou University, Zhengzhou, China

**Keywords:** Oral cancer detection, Salivary gland diseases, Outcomes research, Head and neck cancer, Surgical oncology

## Abstract

Our goals was to evaluate how sentinel lymph node biopsy (SLNB) benefit neck management in patients with parotid cancer. Patients diagnosed with cN0 parotid cancer by preoperative fine needle puncture were prospectively enrolled. The neck status was evaluated by SLNB. If node metastasis was proved by SLNB, a neck dissection of level I–V a was performed, or a wait-and-see policy was conducted for the patient. All related information was extracted and analyzed. Positive SLNB result occurred in 33 (16.7%) patients, and level II metastasis was noted in 100% of the patients. Isolated level III metastasis was noted in 3 (9.1%) patients. During our follow-up with mean time of 49.5 months, 20 patients developed regional recurrence, in which 6 patients had neck dissection as part of their initial management. All the recurrent neck disease was successfully salvaged. Therefore, SLNB was a reliable procedure for neck management in parotid cancer.

## Introduction

Parotid cancer is uncommon and accounts for less than 10% of the head and neck cancers, and cN0 neck management still could not reach agreement. The main focus of the problem is elective neck dissection (END) or wait-and-see policy. Researchers stand for routine END for all parotid cancer based on the following reasons: preoperative grade diagnosis is difficult; END is less invasive, and postoperative functional disorders are less frequent; and lymph node metastasis is frequent in high- and intermediate-grade tumors; the preoperative assessment of lymph node metastasis is limited^1–3^. However, the overall pathologic neck node rate is about 20%^4–6^, routine neck dissection causes the rest patients suffering from overtreatment.

Sentinel lymph node biopsy (SLNB) is widely used in melanoma^7^ and breast cancer^8^ and is more and more popular in the management of head and neck squamous cell carcinoma^9^. However, few authors have analyzed the role of SLNB in treating parotid cancer. Therefore, the current study was the first to evaluate how SLNB benefit cN0 neck management in patients with parotid cancer.

## Results

A total of 198 patients (111 female and 87 male) were enrolled with mean age of 50.2 (range: 19–78) years. The histological tumor stage distribution was: T1 (64 patients), T2 (84 patients), T3 (33 patients), and T4 (17 patients). Negative surgical margin was achieved in all patients. Lymphovascular and perineural invasion was noted in 35 (17.7%) and 29 (14.6%) patients, respectively. The pathological grade distribution was: high grade (21 patients), intermediate grade (31 patients), and low grade (146 patients) (Table 1).

**Table 1:** General Information of the 198 patients.

Variables	N (%)
Age	
≥50	97 (49.0%)
<50	101 (51.0%)
Sex	
Male	87 (43.9%)
Female	111 (56.1%)
Tumor stage	
T1	64 (32.3%)
T2	84 (42.4%)
T3	33 (16.7%)
T4	17 (8.6%)
Neck node stage	
N0	165 (83.3%)
N+	33 (16.7%)
Negative margin	198 (100%)
Perineural invasion	29 (14.6%)
Lymphovascular invasion	35 (17.7%)
Disease grade	
Low	146 (73.7%)
Intermediate	31 (15.7%)
High	21 (10.6%)

Positive SLNB result occurred in 33 (16.7%) patients, and level II metastasis was noted in 100% of the patients, in which level IIa metastasis occurred in 27 patients and level II b metastasis occurred in 7 patients. Level III metastasis was noted in 20 (60.1%) patients, level IV metastasis was noted in 5 (15.2%) patients, level I b and V a metastasis was noted in 1 (3.0%) and 2 (6%) patients, respectively, no patients had level I a metastasis. Moreover, isolated level III metastasis was noted in 3 (9.1%) patients, there was no isolated metastasis in level I, IV, or V a.

**Figure 1:**
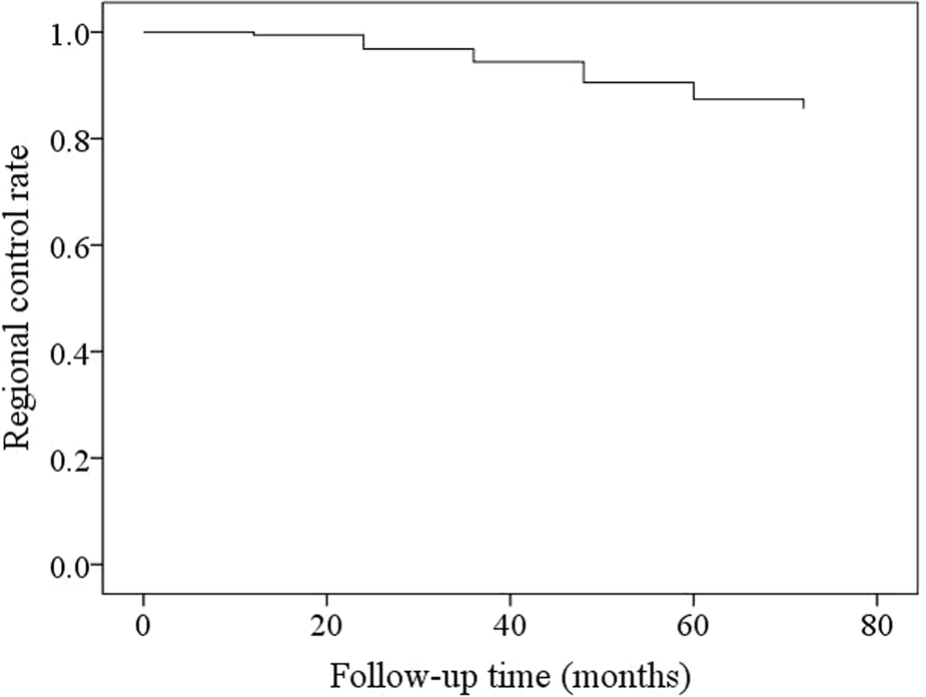
Regional-control rate in patients with parotid cancer.

During our follow-up with mean time of 49.5 (range: 8–90) months, 50 patients received postoperative radiotherapy. 20 patients developed regional recurrence, in which 6 patients had neck dissection as part of their initial management. The 5-year regional control rate was 86% (Fig. 1). All the recurrent neck disease was successfully salvaged.

## Discussion

Parotid cancer was uncommon, but it had a wide range of pathological types, sometimes it was difficult to have a definite pathological diagnosis during frozen section. Currently, the best procedure for neck management remained controversy. However, the most accepted viewpoint was that END was suggested for high grade parotid cancer^1–6^. But as mentioned above, a definite pathological diagnosis could not always be achieved by frozen section, therefore, neck treatment was usually a challenge for the surgeons.

Application of SLNB in parotid cancer patients had been briefly introduced by previous authors^10,11^, but they just reported the technique possibility. We were the first to describe our clinical long term results of SLNB in parotid cancer patients. It was noted that the incidence of positive SLNB result was 16.7%, the finding was a little lower than the occult node metastasis rate in other articles^1,6^, possible explanation was that more low grade cancers existed in our sample, and pathological grade was significantly associated with lymphatic metastasis. It must keep in mind that the above occult metastasis rate was calculated by performing END in all patients, it mean that nearly 80% of the patients were over-treated, and they were in risk for suffering shoulder dysfunction induced by neck dissection^12^. But in current study, no cN0 necks were over-treated, this was the most striking benefit.

Another concern was that whether SLNB cause neck treatment delay. If metastasis nodes were not removed in time, more advanced neck stage would occur, which mean poorer prognosis. Moreover, once there was neck lymphatic metastasis, the survival rate would decreased by an half^13^. In a retrospective report by Kelley *et al*.^1^, 21 patients received END, and 86 patients underwent a wait-and-see policy. The authors described there was no significant difference between the two groups on overall survival, moreover, only 1 patient had neck failure in observation group, compared to 3 patients with END. However, the study was retrospective and patients with END usually had more serious disease at first, therefore, it could not conclude that which procedure was better than the other. In another paper published by Zbaren *et al*.^14^, the authors compared the outcome between END and observation in primary parotid carcinoma, regional recurrence occurred in 7 (17%) patients in the observation group, and in none of the patients with a END, the difference was significant (p = 0.006), all the recurrent neck disease was salvaged by a modified radical or radical neck dissection. The finding was apparently conflicted with ours. The regional recurrence rate was 8.5% in “observation” group, significantly lower than 18.2% in neck dissection group. The difference could be explained by that unlike observation group in other research, the patients undergoing a wait-and-see policy in current study had been screened by SLNB and at low risk for regional recurrence.

Regional recurrence was common for treatment failure in parotid cancer, to achieve satisfactory neck control was one of the main goals. A number of researchers has aimed to explore the potential predictors for recurrence, Elhusseiny *et al*.^15^ enrolled 5446 patients with major salivary gland cancer, the authors reported patients with >18 dissected lymph nodes and 4 positive lymph nodes, and >33.3% lymph nodes ratio were high-risk group patients, similar finding was also reported by Vasan *et al*.^16^ and our previous work^17^, these findings supported the key prognostic value of neck lymph node metastasis, therefore, it was significantly important for the surgeon to find out the patient who has already metastasized at initial treatment. Our favorable oncologic outcome might suggest the protective role of SLNB in neck management. Similarly, Terhaard *et al*.^18^ summarized 565 patients with salivary gland cancer from national register system and reported a 10-year regional control rate of 87%, Matsuda *et al*.^19^ depicted the 5-year locoregional control rate was 89% in patients undergoing surgery plus postoperative radiotherapy. Additionally, as high as 50% of the recurrent neck disease usually could not be salvaged by surgery in squamous cell carcinoma of the head and neck^13^, but it was noted that all the recurrent patients were successfully treated by radical neck dissection partial owing to the reliability of SLNB in neck management in current study. The role of radiation in preventing neck recurrence could not be neglected^20^, Patients with positive SLNB results or high grade cancers were usually suggested for postoperative radiotherapy, it might also be responsible for our favorable outcome.

Most of the metastasis occurred in the level II and III, skip metastasis was rare, and usually developed in level III or IV, the finding was consistent with previous reports^3–6^. The rate of level II b metastasis was 21.2%, it was significantly higher than that in squamous cell carcinoma of the head and neck. Possible explanation was the neighbouring relationship between the two anatomic regions.

In summary, SLNB was a reliable procedure for neck management in parotid cancer. Occult node metastasis usually occurred in level II and III.

## Materials and Methods

The Zhengzhou University institutional research committee approved our study, and all participants signed an informed consent agreement for medical research before initial treatment. All methods were performed in accordance with the relevant guidelines and regulations.

All procedures performed in studies involving human participants were in accordance with the ethical standards of the institutional and/or national research committee and with the 1964 Helsinki declaration and its later amendments or comparable ethical standards.

Patients diagnosed with parotid cancer by preoperative fine needle puncture were prospectively enrolled from January 2010 to December 2016 in our cancer center, they were also required to have cN0 neck disease by preoperative examination including ultrasound, CT and MRI. All related information including age, sex, TNM stage (according to AJCC 2017 classification), disease grade (WHO 2017 classification), and follow-up was extracted and analyzed. Resection of primary foci consisted of total parotidectomy with or without preservation of facial nerve. During SLNB, local anesthesia with light sedation was administered to the patient, and a 99MTc-labelled colloid tracer was injected around the tumor, localization of sentinel lymph nodes was performed using a gamma detection system with a hand-hold probe, then the nodes were biopsied. If node metastasis was proved by SLNB, a neck dissection of level I–V a was performed, or a wait-and-see policy was conducted for the patient.

The Kaplan-Meier method was used to analyze regional control (RC) rate. All statistic analysis was performed by SPSS 20.0, a p < 0.05 was considered to be significant.

## Data Availability

All data generated or analyzed during this study are included in this published article. And the primary data could be achieved from the corresponding author.
